# Effects of Acute Endurance Exercise on Plasma Protein Profiles of Endurance-Trained and Untrained Individuals over Time

**DOI:** 10.1155/2016/4851935

**Published:** 2016-04-30

**Authors:** Marius Schild, Gerrit Eichner, Thomas Beiter, Martina Zügel, Ilke Krumholz-Wagner, Jens Hudemann, Christian Pilat, Karsten Krüger, Andreas M. Niess, Jürgen M. Steinacker, Frank C. Mooren

**Affiliations:** ^1^Department of Sports Medicine, Justus-Liebig University Giessen, Kugelberg 62, 35394 Giessen, Germany; ^2^Mathematical Institute, Justus-Liebig University Giessen, Arndtstraße 2, 35392 Giessen, Germany; ^3^Department of Sports Medicine, University Hospital of Tuebingen, Hoppe-Seyler-Straße 6, 72076 Tuebingen, Germany; ^4^Division of Sport and Rehabilitation Medicine, University Hospital Ulm, Parkstraße 11, 89075 Ulm, Germany

## Abstract

Acute physical exercise and repeated exercise stimuli affect whole-body metabolic and immunologic homeostasis. The aim of this study was to determine plasma protein profiles of trained (EET, *n* = 19) and untrained (SED, *n* = 17) individuals at rest and in response to an acute bout of endurance exercise. Participants completed a bicycle exercise test at an intensity corresponding to 80% of their VO_2max_. Plasma samples were taken before, directly after, and three hours after exercise and analyzed using multiplex immunoassays. Seventy-eight plasma variables were included in the final analysis. Twenty-nine variables displayed significant acute exercise effects in both groups. Seven proteins differed between groups, without being affected by acute exercise. Among these A2Macro and IL-5 were higher in EET individuals while leptin showed elevated levels in SED individuals. Fifteen variables revealed group and time differences with elevated levels for IL-3, IL-7, IL-10, and TNFR2 in EET individuals. An interaction effect could be observed for nine variables including IL-6, MMP-2, MMP-3, and muscle damage markers. The proteins that differ between groups indicate a long-term exercise effect on plasma protein concentrations. These findings might be of importance in the development of exercise-based strategies in the prevention and therapy of chronic metabolic and inflammatory diseases and for training monitoring.

## 1. Introduction

Elucidating the complex processes that determine the human body's ability to adapt to specific training stimuli is crucial to improve athletic performance in elite sports. Moreover, targeted exercise-based intervention programs provide enormous potential to combat the global epidemic increase of chronic metabolic and inflammatory disorders including obesity, metabolic syndrome, and type II diabetes. The systemic and physiological responses to exercise are complex and not well understood and involve a wide range of metabolic, immunological, and hormonal changes [1]. Hereby, the immediate systemic response to acute exercise is known to be closely dependent on the type, duration, and intensity of exercise. However, also the individuals' training status has a major impact on the systemic response to acute exercise, indicating an adaptive response to long-term changes in whole-body repetitive exercise stimuli [2].

There is extensive research dealing with the specific response of different exercise regimes in relation of age, gender, training status, and physical conditions including obesity and diverse chronic inflammatory disorders [3–9]. Most of these studies focus on exercise-induced responses of selected plasma cytokines altered by acute exercise stimuli [10–14]. Besides their roles in mediating immunological responses, specific cytokines have recently emerged to profoundly influence diverse nonimmunological processes, including metabolic functions (e.g., glucose and lipid metabolism), repair and/or prevention of tissue damage, and skeletal muscle remodeling [15–17]. These diverse actions depend on complex and still largely undeciphered crosstalk with various other metabolic and humoral biomolecules that are also released in response to physical activity. These signaling molecules can act synergistically as well as in an independent manner to meet the specific physiological and metabolic demands of the exercising body. The simultaneous detection of a wide range of plasma biomolecules by recently developed multianalyte profiling methods now offers the possibility of generating a more comprehensive picture of the complex systemic responses to exercise [18]. In the present study, an evaluation of at least 90 plasma proteins in trained and untrained individuals at rest and in response to a standardized endurance exercise protocol was conducted. This approach offers the unique opportunity to obtain more comprehensive insights into the systemic response to acute exercise under the precondition of different training statuses. Our analyzed panel included various cytokines and their soluble receptors, as well as acute-phase proteins, hormones, metabolic marker proteins, and muscle damage markers. We hypothesized that regular endurance exercise should be reflected by specific baseline plasma signatures and that acute exercise-provoked plasma variable profiles should display training status dependent response patterns. The determination of protein profiles under different preconditions will contribute to a better understanding of the complex mechanisms of systemic exercise-induced adaptation processes. These findings can be of importance for training monitoring in elite sports and in the development of exercise-based strategies for the prevention and/or therapy of chronic metabolic and inflammatory degenerative diseases. In a previous study, we analyzed skeletal muscle specimen from a subset of these individuals using an off-gel liquid chromatography tandem mass spectrometry (LC-MS/MS) approach. Proteome profiling revealed differences in various metabolic, especially mitochondrial proteins between endurance-trained and untrained individuals under resting conditions and in response to acute exercise [19]. In addition, a subgroup analysis of participants and variables was performed in connection with extracellular DNA traps and cell-free DNA evaluation in plasma [20].

## 2. Methods

### 2.1. Participants and Group Characterization

Healthy male endurance-exercise-trained (EET) athletes as well as untrained/sedentary (SED) individuals were recruited based on training history and aerobic capacity. The inclusion criteria for the EET group (*n* = 19) were regular endurance exercise training of minimum 5 hours per week for at least 5 years before participation and an individual maximal oxygen uptake (VO_2max_) > 57 mL/min/kg body weight (BW). The inclusion criteria for the SED group (*n* = 17) were less than 2 hours of unspecific sports per week and a VO_2max_ < 47 mL/min/kg BW. The anthropometric data for both groups are shown in Table 1. All experimental procedures were approved by the local Ethics Committee of Justus-Liebig University Giessen, the University Hospital of Tuebingen, and the University of Ulm. Each participant underwent a medical screening and had to sign a written consent before the experimental procedure.

### 2.2. Performance Diagnostic

Initially, maximum oxygen uptake was determined by cardiopulmonary exercise testing (MetaLyzer; CORTEX Biophysics, Leipzig, Germany) with an incremental protocol on a bicycle ergometer (Excalibur Sport; Lode, Groningen, Netherlands) to determine the VO_2max_ including capillary blood lactate diagnostics (Biosen; EKF Diagnostics, Magdeburg, Germany) to evaluate the individual performance. Results of the performance diagnostics are shown in Table 1.

### 2.3. Exercise Protocol and Sample Collection

Within one to four weeks after the performance diagnostic, the main exercise protocol was performed. Participants had to abstain from physical activity for 3 days before intervention. After an overnight fast (from 21.00 pm to 08.00 am), participants received standardized breakfast and lunch on the day of acute exercise. On the day before, blood samples were taken at rest (called the “pre” measurements) under the same standardized conditions.

**Figure 1:**
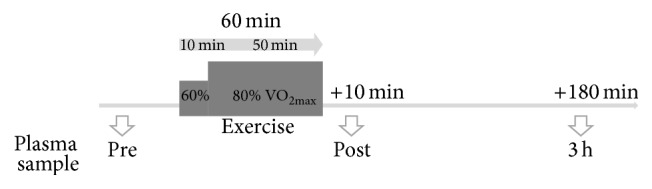
Experimental setup of the study enrollment.

The exercise setup comprised an acute endurance exercise protocol on a bicycle ergometer for 50 min at an intensity (watts) corresponding to 80% of their individual VO_2max_. A 10 min warm-up at 60% of their individual VO_2max_ preceded the endurance trial. Study participants were instructed to drink a defined amount of water corresponding to their body weight at two points in time during the cycling exercise protocol. At ten minutes and at three hours after the acute exercise protocol, further blood samples were collected (called “post” and “3 h,” resp.). A schematic drawing of the study can be found in Figure 1.

### 2.4. Analysis of Whole Blood and Plasma Samples

EDTA blood samples were collected for whole blood cell count and preparation of plasma samples. Complete blood count was accomplished by Synlab Lab Services (Synlab Medical Care Center Kassel GmbH, Kassel, Germany). EDTA tubes for plasma isolation were kept on ice and centrifuged at 1600 g for 10 min within 30 min after collection (Universal 320R, Hettich centrifuges, Tuttlingen, Germany). Plasma supernatant was collected, immediately frozen in liquid nitrogen, and stored at −80°C.

Quantification of 90 analytes in EDTA plasma samples was performed at Myriad Rules-Based Medicine (Myriad RBM, Austin, USA) using HumanMAP v. 1.6 of their Luminex-based multiplex immunoassays as described by the vendor. Only variables that could be detected in at least 80% of the plasma samples were taken into account for further statistical analysis, using a procedure for multiplex measurements [21, 22]. This procedure resulted in the inclusion of 78 of the 90 available variables of HumanMAP v. 1.6. Samples of the included variables that had no detectable value were set at half of the lowest measured value for the corresponding variable. To exclude biased measures due to plasma volume shift, changes in plasma concentrations were calculated from haemoglobin and haematocrit values, and concentrations of postexercise variables were corrected as described by Kraemer and Brown [23] and accomplished previously [20].

In addition, granulocyte-colony stimulating factor (G-CSF) was chosen for separate analysis on the basis of recent findings in its role in exercise delayed neutrophil apoptosis and progenitor cell mobilization [14, 24]. G-CSF concentration in plasma samples was measured using a high sensitive enzyme-linked immunosorbent assay (hsELISA) kit (R&D Systems, Minneapolis, USA) according to the manufacturers' instructions. The concentration of G-CSF was calculated using* MikroWin 2000* v. 4.34 (Mikrotek Labor Systems, Overath, Germany). Mean ± standard deviation (SD) and median with the 1. and 3. quartile for all included variables can be found in Supplemental Table S1 in Supplementary Material available online at http://dx.doi.org/10.1155/2016/4851935.

### 2.5. Statistical and Bioinformational Analysis

Physiological data were statistically analyzed using Wilcoxon's rank sum test for independent samples for differences between groups and presented as mean ± SD and median with the 1. and 3. quartile. Plasma and whole blood data were statistically analyzed using a nonparametric two-factorial analysis of variance (ANOVA) for longitudinal data to assess the main effects of time and group and their interaction effects [25]. The chosen nonparametric method allows us to analyze the data without the need of normality or data transformations [25]. The analysis is based on the so-called relative treatment effects (RTEs) and data are presented as RTE with the corresponding 95% confidence intervals (CI). Briefly described, a RTE has a value between 0 and 1 and indicates the probability that a measurement in one group at a given time point is larger than a value of this variable in any other combination of group and time. Keeping this in mind, a RTE > 0.5 means that the EET group tends to have larger values than the SED group, whereas a RTE < 0.5 must be interpreted as tendency towards lower values in EET group. For more details see [25–27]. When a significant main effect was observed, appropriate post hoc pairwise comparison for different time points (repeated nonparametric one-factorial ANOVA for longitudinal data with time as factor) or between groups (Wilcoxon's rank sum test) was performed. Correlation analysis in the EET group for plasma variables that differ significantly at baseline between groups with their corresponding VO_2max_ was performed using Spearman's rank correlation. For all analysis a *p* value < 0.05 was considered to be significant. Whenever necessary to account for multiple testing, Holm's correction method for *p* values was performed [28]. Hierarchical cluster analysis of the RTEs was performed using Ward's method. In addition, the scores of the area under the curve with respect to the increase (AUC_I_) were calculated for all plasma variables and compared by Student's *t*-test or Wilcoxon's rank sum test when appropriate as described previously [29]. AUC_I_ scores for significant different variables are shown in Supplemental Table S2. The statistics software R, version 3.0.2 [30], and the packages nparLD [26] and nparcomp [31], GraphPad Prism 5.0 (GraphPad Software, La Jolla, USA), JMP Pro 10.0.2 (SAS Institute, Cary, USA), and IBM SPSS v. 21 (IBM Corp., Armonk, USA) were used for statistical analysis.

## 3. Results

### 3.1. Acute Endurance Exercise Trial

The acute endurance exercise at 80% VO_2max_ in watts led to a significantly higher workload in the EET group (*p* < 0.001). There were no differences in lactate levels and heart rate (HR) between the two groups during the one-hour-lasting exercise (Table 1). Lactate values started to increase significantly after half of the main exercise protocol was completed and remained elevated until the end of the exercise trial, with no significant differences between groups (*p* < 0.001).

### 3.2. Leukocyte Counts

Total leukocytes and neutrophil numbers significantly increased over time until 3 h after exercise. Lymphocytes and monocytes increased directly after exercise. At three hours after exercise, lymphocytes had decreased to baseline, whereas monocytes remained elevated (Table 2).

### 3.3. Plasma Variable Results

#### 3.3.1. Response of Plasma Variables over Time Independent of Group Affiliation

**Figure 2:**
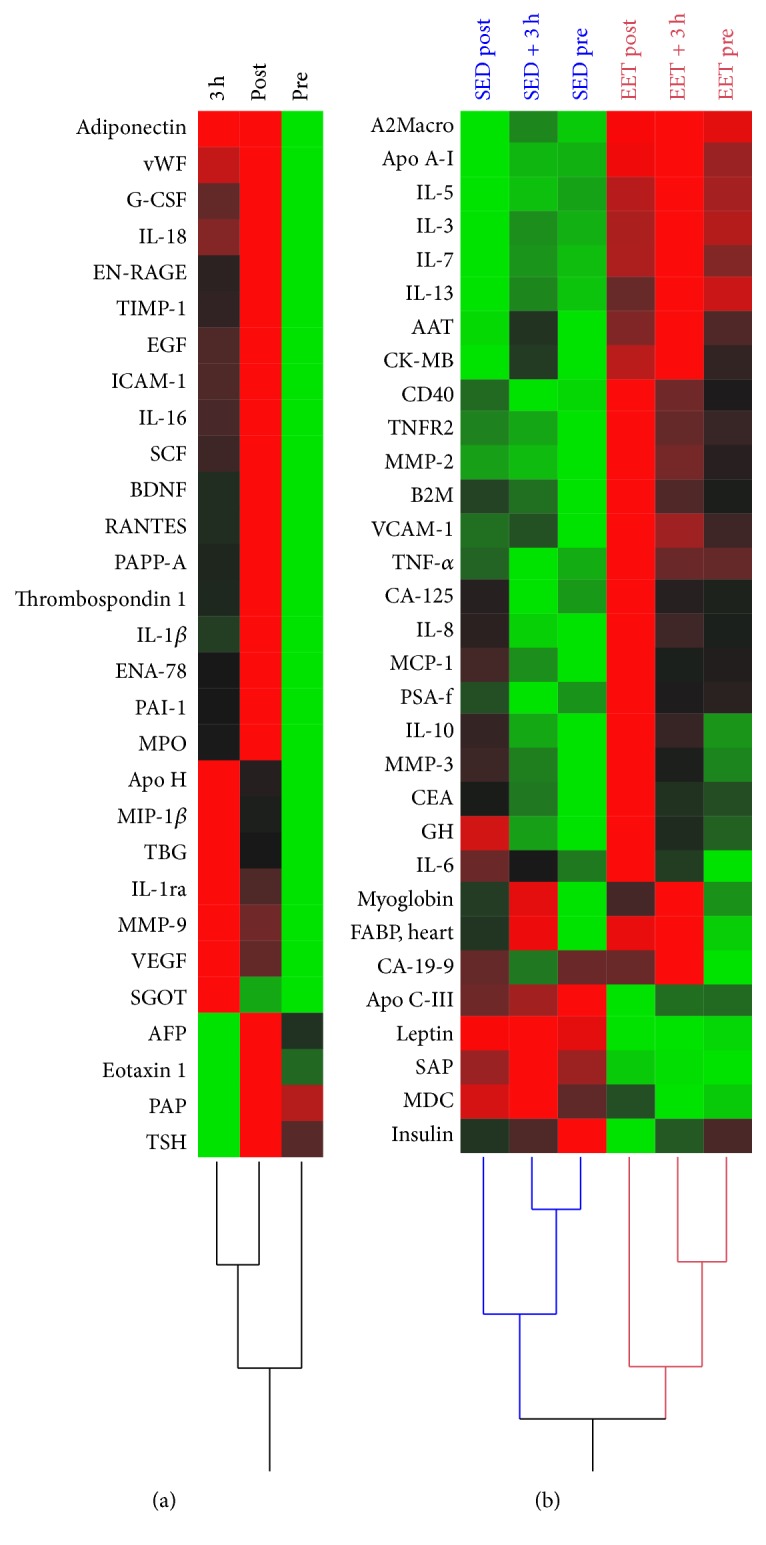
Hierarchical cluster analysis of significant variables with (a) main effect time point and (b) group, group and time, and interaction effect at each time point between EET and SED individuals using Ward's method. Differences are represented by the computed RTEs. Red colour representing higher and green lower RTEs. EET: endurance-exercise-trained; SED: untrained/sedentary.

Twenty-nine of the 78 included variables showed an effect in response to the acute exercise intervention without differences between the EET and SED group (Table 3(a)). Marked responses to exercise were observed for the proinflammatory and chemoattractant interleukins interleukin- (IL-) 1 beta (IL-1*β*) and IL-16 directly after exercise, whereas the anti-inflammatory IL-1*β* antagonist IL-1 receptor antagonist (IL-1ra) showed a delayed increase 3 h after exercise. Matrix metalloproteinase-9 (MMP-9) also increased over time, whereas its antagonist, the tissue inhibitor of metalloproteinase-1 (TIMP-1), showed an immediate response pattern and declined to baseline values 3 h after exercise. Other inflammation-associated analytes, including the neutrophil-derived extracellular newly identified receptor for advanced glycation end-products binding protein (EN-RAGE) as well as myeloperoxidase (MPO) and the chemokine epithelial-derived neutrophil-activating protein 78 (ENA-78) and the T-cell-specific protein RANTES (RANTES), also increased markedly in response to exercise and remained elevated at the 3 h time point. We could further observe increased levels of growth factors such as brain-derived neurotrophic factor (BDNF), granulocyte-colony stimulating factor (G-CSF), stem cell factor (SCF), and vascular endothelial growth factor (VEGF) directly after exercise. G-CSF, SCF, and VEFG remained elevated 3 h after exercise in contrast to BDNF. The same pattern applies for plasma levels of adhesive glycoproteins thrombospondin 1 and von Willebrand factor (vWF) which also increased immediately after exercise and remained elevated. The RTEs of all 29 variables that display a significant time effect are clustered in Figure 2(a).

#### 3.3.2. Differences between Groups in Plasma Variables without Acute Exercise Effects

Seven plasma variables differed between groups at baseline but were not affected by the exercise intervention (Table 3(b)). In the EET group, elevated levels could be observed for the protease inhibitor protein alpha-2-macroglobulin (A2Macro), Apolipoprotein (Apo) A-I (Apo A-I), a major component of high-density lipoproteins (HDL), and T-helper (Th) type 2 and hematopoietic cytokine IL-5. In contrast, significant higher baseline values in the SED group were evident for the very low density lipoprotein component Apo C-III, the chemokine macrophage-derived chemokine (MDC), the acute-phase protein serum amyloid P-component (SAP), and the adipokine leptin.

#### 3.3.3. Variables Which Revealed Group and Time Point Differences between Groups

Fifteen of the analyzed variables showed significant main effects for both group and time (Table 3(c)). We found group differences for the chemoattractant proteins IL-8 and monocyte chemotactic protein-1 (MCP-1) with higher levels in the EET group. Both chemokines increased directly after exercise in both groups while MCP-1 already displayed elevated baseline levels in the EET group. Likewise, baseline values of the anti-inflammatory IL-10 differed between groups with increased levels in the EET group. In response to exercise cytokine IL-10 increased in both the EET and SED group but remained elevated only in the EET group 3 h after exercise. Th2-type cytokines IL-3 and IL-13 revealed no post hoc time effects but differed markedly between groups with higher levels in the EET group. Exercise-provoked increases in tumor necrosis factor-alpha (TNF-*α*) levels could only be observed in the EET group directly in response to exercise. Concordantly, plasma levels of tumor necrosis factor receptor 2 (TNFR2) were significantly higher in the EET group—already at rest—and an immediate increase in response to exercise could only be observed in the EET group. The protease inhibitor protein alpha-1-antitrypsin (AAT) was elevated in the EET group compared to the SED group with exercise dependent time effects in the SED group 3 h after exercise. We further found myoglobin, an early and sensitive marker for muscle membrane damage, to be elevated in the EET group at rest. An exercise-provoked release over time could be observed in both groups, with increasing levels at 3 h after exercise. Similarly, the insulin antagonist anabolic growth hormone (GH) differed between groups with significantly elevated levels at rest in the EET group. GH immediately increase in response to exercise and remained elevated 3 h after exercise in both groups. In contrast, elevated levels of insulin could be detected in the SED group compared to the EET group. Insulin dropped immediately after exercise in both the EET and SED group but remained decreased at 3 h after exercise only in the EET group.

#### 3.3.4. Variables That Differ Significantly in Time Courses between the EET and SED Group

Nine of the analyzed variables exhibited a significant interaction effect between group and time (Table 3(d)). Extracellular matrix proteins MMP-2 and MMP-3 revealed different time curves between EET and SED group. An effect of exercise for MMP-2 could only be observed in the EET group and MMP-3 remained elevated only in the EET group at 3 h after exercise. Both MMP-2 and MMP-3 were significantly enhanced in the EET group immediately after exercise. The pleiotropic cytokine IL-6 increased in response to exercise in both groups. The increase of IL-6 was more pronounced in the EET group immediately after exercise but post hoc tests revealed no significant differences between groups (*p* = 0.07). Interaction effects could also be observed for the muscle damage markers creatine kinase MB (CK-MB) and heart-type fatty acid-binding protein (FABP). FABP increased directly after exercise with higher levels in the EET group and remained elevated in both groups. CK-MB was significantly higher at all three sampling points in the EET group. However, a delayed increase also became apparent in the SED group at 3 h after exercise.

The nonparametric analysis revealed no significant main or interaction effects of time and groups for 19 variables. These variables can be found in Supplemental Table S3.

RTEs for significant variables with group, group and time, and interaction (group by time) effect for EET and SED groups (Tables 3(b)–3(d)) are clustered in Figure 2(b). Plasma variables that differ significantly between the EET and SED group at baseline (pre) are displayed in Table 4. Correlation analyses of baseline plasma variable values with VO_2max_ for the EET group revealed a significant positive correlation for Apo A-I (*r* = 0.66, *p* = 0.03) and a negative correlation for leptin (*r* = −0.68, *p* = 0.02). All correlations can be found in Supplemental Table S4.

## 4. Discussion

In the present study, we compared the plasma protein profiles of endurance-exercise-trained (EET) and sedentary (SED) individuals at rest and the effects of an acute bout of intensive endurance exercise on these circulating blood variables over time in both groups.

### 4.1. Interplay of Immune System and Metabolic Regulation

The systemic response to acute exercise relies on coordinated crosstalk of body tissues to maintain metabolic and immune homeostasis [32–34]. The immune system has to preserve an adequate immune function towards exogenous pathogens while maintaining self-tolerance to endogenous components that are released during exercise from skeletal muscle and other tissues. The acute endurance exercise increased circulating levels of muscle cell damage and permeability markers such as myoglobin, CK-MB, and FABP illustrating the need for an adequate self-tolerance in response to strenuous exercise. Here, an increase in muscle damage markers could be observed, especially in the EET group. Key cytokines IL-3, IL-5, IL-7, and IL-13, which have the ability to skew T-cell cytokine secretion to T-helper (Th) type 2 responses, stimulate dendritic cell differentiation, and maintain T-cell homeostasis [35, 36], showed significant group and time effects with higher levels in the EET group. These effects could also be observed for immunosuppressive mediators IL-10 and TNFR2 [37, 38]. These findings indicate that regular exercise provokes acute as well as long-term changes in Th2-type (IL-5, IL-6, and IL-13) [39] and regulatory T-cell (Treg) (IL-10) [37] cytokines with more pronounced effects in trained athletes compared to sedentary participants.

Despite their role in inflammation, cytokines also function as mediators of energy metabolism signaling. Immune function and metabolic regulation are tightly interconnected when it comes to exercise-induced alterations. High IL-10 levels, for example, have been reported to promote insulin sensitivity in humans and protect against insulin resistance by diminishing TNF mediated intracellular signaling in adipocytes [40]. Apo A-I, a major component of high-density lipoprotein (HDL) particles, has been shown to have anti-inflammatory effects on blood cells and adipocytes [41] but is also postulated to improve cellular energy glucose metabolism and increase glucose uptake by muscle cells [42]. Here, we could observe a positive correlation between Apo A-I and the endurance capacity represented by VO_2max_ in the EET group under resting conditions. Apo A-I was not affected by acute exercise but showed a significant group effect with higher levels in the EET group. In line with this, the LC-MS/MS skeletal muscle analysis revealed elevated levels of key enzymes of the oxidative energy metabolism in the endurance-trained muscle and, therefore, the ability for enhanced muscular glucose utilization [19]. During intense endurance exercise, glucose is the major fuel for the exercising muscle mobilized from muscle and liver glycogen stores. The observed decrease in blood insulin in response to exercise, especially in the EET group, enables the mobilization of glucose for energy expenditure. One key player in the acute metabolic signaling is the pleiotropic cytokine IL-6 that is locally secreted by the contracting skeletal muscle [43] and found to be elevated in circulation in response to exercise [17]. In the present study, the highest values of IL-6 for both groups were measured immediately after exercise with a more pronounced effect in the EET group. IL-6 has been demonstrated to provide metabolic effects, like enhanced glycogenolysis and fatty acid oxidation [15], which correspond to the elevated abundance of mitochondrial enzymes of the tricarboxylic acid and oxidative phosphorylation in the endurance-trained skeletal muscle [19] and, therefore, the possibility of utilizing more substrate for energy expenditure. IL-6 knockout mice showed an impaired carbohydrate and lipid metabolism and, in addition, were insensitive towards the metabolic effects of leptin despite elevated circulating plasma leptin level [44]. Our study revealed significantly lower plasma leptin levels in the EET group but no impact of acute exercise. In addition, within the endurance-trained individuals, leptin was negatively correlated with the VO_2max_ at baseline. A reduction in blood leptin levels has recently been implicated with both an improved endurance training status and changes in body composition [45]. In addition to its metabolic functions, leptin is considered to have proinflammatory activity and stimulates Th1-type cytokines [46] whereas the lower leptin levels in the EET group are in line with the abovementioned shift towards Th2-type cytokines in trained individuals. In contrast, the proinflammatory cytokine TNF-*α* and the chemokines IL-8 and MCP-1 revealed group and time effects and were also increased in the EET group after exercise. In the context of exercise, one might speculate about metabolic functions of the rather proinflammatory cytokines. MCP-1, for example, is suggested to play a role in energy metabolism. MCP-1 knockout mice showed increased insulin levels accompanied with impaired glucose utilization [47].

### 4.2. Regulatory Interactions

Remarkably, the protease inhibitors AAT and A2Macro were found to be elevated in the EET group whereas AAT also increased 3 h after exercise in the SED group. AAT serves as an inhibitor of neutrophil-derived elastase [48] and might reflect a response to exercise-provoked neutrophilia and increased elastase release to limit its adverse effects [49]. Similarly, A2Macro, a well-known inhibitor of proteinases, released by neutrophils in the inflammatory process, protects human tissue from collateral damage caused by unregulated proteolytic activity [50]. Moreover, A2Macro binds to several cytokines, including TNF-*α*, IL-1*β*, and IL-6, and modulates their biological activity. The TNF-*α* complex may be removed by complex formation with A2Macro from circulation and the biological activity of IL-1*β* is inhibited by A2Macro [50]. In contrast, IL-6 activity has been reported to remain partly active, and IL-6 is protected from degradation and available for its target cells when binding to A2Macro [50]. Supportive evidence comes from the regulation of MMP-2 and MMP-3 in response to exercise with significantly higher levels in the EET group. Despite their role in the degradation of extracellular matrix proteins and tissue remodeling, MMPs influence inflammatory processes through their functional inactivation of cytokines and chemokines like IL-1*β* [51, 52]. Therefore, protease inhibitors and MMPs might have the potential to diminish an excessive proinflammatory reaction in response to exercise. Accordingly, we suggest that the trained organism initiates long-term adaptive processes to cope with exercise-induced inflammatory and metabolic stress, thus allowing performances at higher intensities without adverse effects. A deeper understanding of exercise-induced alterations offers the possibility of prescribing customized exercise as medicine in metabolic and inflammatory disorders like obesity, metabolic syndrome, and type II diabetes.

## 5. Concluding Remarks

In the present study, we could demonstrate that acute strenuous endurance exercise has differential effects on circulating blood variables, depending on the athlete's training status. While a wide range of variables responded simultaneously to exercise in sedentary and endurance-trained individuals, we also identified plasma proteins that differ between groups in response to acute exercise. In addition, the analysis revealed plasma proteins that were not affected by acute exercise but differed between groups at baseline, indicating long-term homeostatic adaptations caused by regular endurance exercise training. As a consequence of the systemic and cellular adaptation to inflammatory and metabolic stress evoked by regular exercise, trained athletes are capable of performing at absolute higher workloads. To achieve a similar systemic burden in both groups, a standardized relative workload protocol was chosen (indicated by similar lactate and HR values) that allows a physiological comparison between groups. In consequence, the EET group performed at a higher absolute workload and it should be considered that postexercise plasma levels might also be affected by differences in exercise intensity of both groups.

We believe that our analysis provides meaningful new insights into the effects of endurance exercise on metabolic and inflammatory signaling and homeostasis. Systemic blood variables that are capable of monitoring exercise adaptation are of scientific value not only to improve athletic performance in elite sports but also to develop exercise-based intervention programs in the prevention and treatment of chronic metabolic and inflammatory diseases.

## Supplementary Material

The Supplementary Material comprises in Table S1 the mean ± SD, median with the 1. and 3. quartile for all in the statistical analysis included variables, the AUC_I_ scores for significant different variables are shown in Table S2, Table S3 contains all variables that showed no significant main effect and in Table S4 all performed correlations for the EET group with their corresponding VO_2max_ can be found for plasma variables that differed significantly at baseline between groups.

## Figures and Tables

**Table 1:** The anthropometric data and performance results of the entrance examination and endurance exercise of EET and SED individuals. Shown are mean ± SD and in parentheses the 1. quartile, median, and the 3. quartile. ^*∗∗∗*^ *p* < 0.001 indicates significant differences between endurance-exercise-trained (EET) athletes and untrained/sedentary (SED) individuals.

	EET (*n* = 19)	SED (*n* = 17)
*Anthropometric data*		
Age (years)	25 ± 2.9 (24, 26, 27)	24 ± 3.5 (21, 22, 28)
Weight (kg)	73.9 ± 8.3 (70, 73, 77)	77.9 ± 12.4 (69, 76, 84)
Height (kg)	180.4 ± 6.5 (174, 181, 186)	177.9 ± 5.5 (174, 176, 180)
BMI	22.5 ± 1.6 (21, 23, 24)	24.7 ± 4.1 (22, 24, 27)
*Entrance examination *		
VO_2max_ [mL/min/kg]^*∗∗∗*^	65.6 ± 6.7 (61, 66, 71)	40.5 ± 6.1 (39, 41, 45)
Maximum performance [watts]^*∗∗∗*^	339.5 ± 45.9 (307, 333, 387)	206.4 ± 32.4 (202, 217, 225)
Maximum performance [watts/kg]^*∗∗∗*^	4.6 ± 0.6 (4.0, 4.5, 5.0)	2.7 ± 0.5 (2.5, 2.8, 3.1)
Maximum lactate [mmol/L]	12 ± 2.0 (11, 12, 13)	10.4 ± 3.2 (7, 10, 13)
Maximum HR [beats/min]	188.9 ± 10.0 (183, 191, 196)	188.7 ± 9.3 (182, 188, 197)
Performance at IAT [watts/kg]^*∗∗∗*^	2.9 ± 0.5 (2.5, 2.7, 3.3)	1.4 ± 0.4 (1.2, 1.6, 1.7)
Lactate at IAT [mmol/L]	2.4 ± 0.4 (2.0, 2.4, 2.7)	2.5 ± 0.5 (2.1, 2.5, 2.7)
*Exercise performance*		
Performance at 80% VO_2max_ [watts]^*∗∗∗*^	254.1 ± 45.4 (227, 240, 280)	156.4 ± 32.0 (150, 167, 175)
Average lactate [mmol/L]	6.7 ± 3.1 (4.1, 5.9, 8.9)	6.1 ± 2.9 (3.8, 4.9, 7.1)
HR at end [beats/min]	178.8 ± 12.4 (168, 178, 189)	177.4 ± 8.2 (170, 175, 184)

BMI = Body Mass Index; HR = heart rate; IAT = individual anaerobic threshold; VO_2max_ = maximal oxygen consumption.

**Table 2:** Leukocytes, neutrophils, lymphocytes, and monocytes counts are shown in cells/*μ*L. Displayed is the median with the 1. and 3. quartile in parentheses. ^*∗∗*^ *p* < 0.01 and ^*∗∗∗*^ *p* < 0.001 indicate significant differences from baseline.

Variable	Median (1. quartile; 3. quartile)		
	Pre	Post	3 h
Leukocytes	5080 (4030; 5755)	6209 (5518; 7645)^*∗∗∗*^	8998 (7496; 10565)^*∗∗∗*^
Neutrophils	2614 (2164; 3343)	3556 (2954; 4606)^*∗∗∗*^	6427 (5448; 8472)^*∗∗∗*^
Lymphocytes	1672 (1397; 1850)	2097 (1810; 2456)^*∗∗∗*^	1561 (1350; 1773)
Monocytes	378 (299; 439)	478 (375; 602)^*∗∗*^	501 (397; 610)^*∗∗∗*^

**(a):** 

Variable	RTE (CI)		
	Pre	Post	3 h
Adiponectin	0.47 (0.45; 0.5)	0.51 (0.5; 0.53)	0.51 (0.49; 0.53)
AFP	0.49 (0.46; 0.52)	0.56 (0.52; 0.6)^*∗*^	0.45 (0.42; 0.48)
Apo H	0.46 (0.43; 0.50)	0.50 (0.47; 0.54)	0.53 (0.50; 0.56)^*∗∗∗*^
BDNF	0.44 (0.41; 0.47)	0.57 (0.55; 0.6)^*∗∗∗*^	0.49 (0.46; 0.53)
EN-RAGE	0.25 (0.21; 0.30)	0.72 (0.67; 0.76)^*∗∗∗*^	0.53 (0.49; 0.57)^*∗∗∗*^
Eotaxin 1	0.48 (0.44; 0.52)	0.56 (0.51; 0.61)	0.46 (0.40; 0.51)
EGF	0.46 (0.42; 0.49)	0.53 (0.5; 0.56)^*∗*^	0.51 (0.48; 0.54)
ENA-78	0.44 (0.42; 0.47)	0.56 (0.53; 0.59)^*∗∗∗*^	0.50 (0.47; 0.52)^*∗*^
G-CSF	0.40 (0.35; 0.46)	0.57 (0.54; 0.6)^*∗∗∗*^	0.53 (0.49; 0.57)^*∗*^
ICAM-1	0.46 (0.42; 0.49)	0.53 (0.5; 0.57)^*∗*^	0.51 (0.48; 0.54)
IL-1*β*	0.39 (0.34; 0.46)	0.64 (0.57; 0.69)^*∗∗∗*^	0.47 (0.41; 0.54)
IL-1ra	0.42 (0.37; 0.47)	0.52 (0.46; 0.59)	0.56 (0.50; 0.61)^*∗∗*^
IL-16	0.28 (0.25; 0.32)	0.67 (0.64; 0.70)^*∗∗∗*^	0.55 (0.53; 0.57)^*∗∗∗*^
IL-18	0.46 (0.43; 0.49)	0.52 (0.49; 0.56)	0.51 (0.48; 0.55)^*∗*^
MIP-1*β*	0.45 (0.41; 0.48)	0.50 (0.47; 0.53)	0.56 (0.52; 0.60)^*∗∗*^
MMP-9	0.27 (0.23; 0.31)	0.58 (0.53; 0.61)^*∗∗∗*^	0.66 (0.61; 0.70)^*∗∗∗*^
MPO	0.34 (0.29; 0.39)	0.67 (0.61; 0.71)^*∗∗∗*^	0.50 (0.45; 0.55)^*∗∗∗*^
PAI-1	0.46 (0.43; 0.49)	0.54 (0.51; 0.57)^*∗∗*^	0.50 (0.46; 0.54)
PAPP-A	0.41 (0.35; 0.48)	0.60 (0.55; 0.66)^*∗∗∗*^	0.49 (0.43; 0.54)
PAP	0.56 (0.53; 0.6)	0.58 (0.52; 0.63)	0.36 (0.31; 0.41)^*∗∗∗*^
SGOT	0.45 (0.40; 0.51)	0.46 (0.41; 0.52)	0.58 (0.53; 0.63)^*∗*^
SCF	0.45 (0.42; 0.48)	0.54 (0.51; 0.58)^*∗∗∗*^	0.51 (0.48; 0.55)^*∗*^
RANTES	0.44 (0.41; 0.47)	0.57 (0.54; 0.59)^*∗∗∗*^	0.49 (0.47; 0.52)^*∗*^
Thrombospondin 1	0.42 (0.39; 0.46)	0.59 (0.56; 0.62)^*∗∗∗*^	0.49 (0.45; 0.52)^*∗*^
TSH	0.53 (0.48; 0.57)	0.58 (0.54; 0.63)	0.39 (0.35; 0.43)^*∗∗∗*^
TBG	0.46 (0.42; 0.50)	0.50 (0.47; 0.53)	0.54 (0.51; 0.58)^*∗*^
TIMP-1	0.43 (0.39; 0.48)	0.56 (0.51; 0.61)^*∗∗*^	0.51 (0.46; 0.55)
VEGF	0.40 (0.37; 0.44)	0.53 (0.5; 0.57)^*∗∗∗*^	0.57 (0.52; 0.61)^*∗∗∗*^
vWF	0.32 (0.26; 0.40)	0.60 (0.54; 0.66)^*∗∗∗*^	0.58 (0.5; 0.64)^*∗∗∗*^

AFP = Alpha Fetoprotein; Apo H = Apolipoprotein H; BDNF = brain-derived neurotrophic factor; EN-RAGE: extracellular newly identified receptor for advanced glycation end-products binding protein; EGF = Epidermal Growth Factor; ENA-78 = epithelial-derived neutrophil-activating protein 78; G-CSF = granulocyte-colony stimulating factor; ICAM-1 = Intercellular Adhesion Molecule 1; IL-1*β* = interleukin-1 beta; IL-1ra = interleukin-1 receptor antagonist; IL-16 = interleukin-16; IL-18 = interleukin-18; MIP-1*β* = macrophage inflammatory protein-1 beta; MMP-9 = matrix metalloproteinase-9; MPO = myeloperoxidase; PAI-1 = Plasminogen Activator Inhibitor-1; PAPP-A = Pregnancy Associated Plasma Protein-A; PAP = Prostatic Acid Phosphatase; RANTES = regulated upon activation normal T-cell expressed and secreted; SGOT = Serum Glutamic Oxaloacetic Transaminase; SCF = stem cell factor; TSH = Thyroid Stimulating Hormone; TBG = Thyroxine Binding Globulin; TIMP-1 = tissue inhibitor of metalloproteinase-1; VEGF = vascular endothelial growth factor; vWF = von Willebrand factor.

**(b):** 

Variable	RTE (CI)
A2Macro^$$^	0.71 (0.60; 0.82)
Apo A-I^$^	0.71 (0.60; 0.81)
Apo C-III^$^	0.32 (0.21; 0.43)
IL-5^$$^	0.77 (0.67; 0.87)
Leptin^$$$^	0.09 (0.03; 0.14)
MDC^$^	0.32 (0.22; 0.43)
SAP^$$$^	0.22 (0.13; 0.31)

A2Macro = alpha-2-macroglobulin; APO A-I = Apolipoprotein A-I; Apo C-III = Apolipoprotein C-III; IL-5 = interleukin-5; MDC = macrophage-derived chemokine; SAP = serum amyloid P-component.

**(c):** 

Variable	Group	RTE (CI)		
		Pre	Post	3 h
AAT	EET	0.55 (0.45; 0.65)	0.59 (0.48; 0.68)	0.66 (0.56; 0.74)
	SED^$$^	0.35 (0.27; 0.43)	0.35 (0.25; 0.47)	0.46 (0.38; 0.55)^*∗∗*^

CA-125	EET	0.48 (0.38; 0.59)	0.74 (0.63; 0.81)^*∗∗∗*^	0.52 (0.42; 0.61)
	SED^$^	0.38 (0.29; 0.48)	0.52 (0.42; 0.62)^*∗*^	0.33 (0.25; 0.44)

CD40	EET	0.50 (0.4; 0.6)	0.68 (0.59; 0.76)^*∗∗∗*^	0.59 (0.49; 0.68)^*∗*^
	SED^$^	0.38 (0.29; 0.49)	0.44 (0.33; 0.55)	0.38 (0.28; 0.49)

GH	EET	0.37 (0.31; 0.43)	0.85 (0.81; 0.87)^*∗∗∗*^	0.45 (0.4; 0.51)^*∗*^
	SED^$$^	0.22 (0.17; 0.29)	0.79 (0.74; 0.83)^*∗∗∗*^	0.29 (0.22; 0.39)^*∗*^

Insulin	EET	0.57 (0.48; 0.65)	0.30 (0.23; 0.39)^*∗∗∗*^	0.42 (0.31; 0.53)^*∗*^
	SED^$^	0.71 (0.59; 0.8)	0.46 (0.34; 0.58)^*∗∗*^	0.57 (0.45; 0.68)

IL-3	EET	0.63 (0.54; 0.71)	0.62 (0.53; 0.7)	0.68 (0.59; 0.75)
	SED^$$$^	0.34 (0.26; 0.44)	0.31 (0.23; 0.4)	0.37 (0.29; 0.46)

IL-7	EET	0.59 (0.5; 0.67)	0.62 (0.53; 0.69)	0.66 (0.57; 0.74)^*∗∗*^
	SED^$$^	0.36 (0.27; 0.47)	0.34 (0.26; 0.44)	0.39 (0.3; 0.48)

IL-8	EET	0.48 (0.39; 0.58)	0.73 (0.66; 0.79)^*∗∗∗*^	0.56 (0.46; 0.64)
	SED^$$^	0.33 (0.23; 0.45)	0.53 (0.41; 0.63)^*∗∗*^	0.34 (0.26; 0.43)

IL-10	EET	0.37 (0.29; 0.46)	0.81 (0.74; 0.86)^*∗∗∗*^	0.56 (0.46; 0.65)^*∗∗*^
	SED^$$^	0.32 (0.24; 0.41)	0.56 (0.45; 0.65)^*∗∗*^	0.36 (0.27; 0.46)

IL-13	EET	0.60 (0.51; 0.69)	0.55 (0.46; 0.64)	0.63 (0.53; 0.71)
	SED^$^	0.39 (0.3; 0.49)	0.38 (0.29; 0.48)	0.42 (0.32; 0.52)

MCP-1	EET	0.52 (0.42; 0.61)	0.77 (0.69; 0.83)^*∗∗∗*^	0.48 (0.4; 0.57)
	SED^$$^	0.28 (0.21; 0.37)	0.57 (0.44; 0.69)^*∗∗∗*^	0.35 (0.25; 0.47)

Myoglobin	EET	0.27 (0.21; 0.35)	0.58 (0.51; 0.64)^*∗∗∗*^	0.79 (0.74; 0.82)^*∗∗∗*^
	SED^$^	0.17 (0.14; 0.23)	0.41 (0.33; 0.50)^*∗∗∗*^	0.76 (0.66; 0.83)^*∗∗∗*^

TNF-*α*	EET	0.56 (0.47; 0.64)	0.64 (0.55; 0.71)^*∗*^	0.56 (0.47; 0.65)
	SED^$^	0.40 (0.3; 0.51)	0.44 (0.35; 0.53)	0.38 (0.29; 0.48)

TNFR2	EET	0.54 (0.45; 0.63)	0.74 (0.65; 0.8)^*∗∗∗*^	0.60 (0.52; 0.68)
	SED^$$$^	0.32 (0.24; 0.42)	0.38 (0.31; 0.47)	0.36 (0.28; 0.45)

VCAM-1	EET	0.53 (0.44; 0.63)	0.66 (0.57; 0.73)^*∗*^	0.60 (0.51; 0.69)
	SED^$^	0.33 (0.26; 0.42)	0.41 (0.32; 0.51)^*∗*^	0.43 (0.34; 0.53)^*∗*^

AAT = alpha-1-antitrypsin; CA-125 = Cancer Antigen 125; CD40 = CD 40 antigen; GH = growth hormone; IL-3 = interleukin-3; IL-7 = interleukin-7; IL-8 = interleukin-8; IL-10 = interleukin-10; IL-13 = interleukin-13; MCP-1 = monocyte chemotactic protein-1; TNF-*α* = tumor necrosis factor-alpha; TNFR2 = tumor necrosis factor receptor 2; VCAM-1 = Vascular Cell Adhesion Molecule-1.

**(d):** 

Variable	Group	RTE (CI)		
		Pre	Post	3 h
B2M	EET	0.48 (0.39; 0.57)	0.82 (0.75; 0.86)^*∗∗∗*^	0.60 (0.51; 0.69)^*∗*^
	SED^§/$$$^	0.26 (0.20; 0.34)^$^	0.42 (0.34; 0.52)^*∗∗*/$$$^	0.37 (0.29; 0.46)^$^

CA-19-9	EET	0.44 (0.34; 0.55)	0.52 (0.42; 0.62)	0.54 (0.45; 0.63)
	SED^§^	0.52 (0.41; 0.63)	0.52 (0.42; 0.62)	0.46 (0.36; 0.57)

CEA	EET	0.47 (0.38; 0.57)	0.67 (0.58; 0.74)^*∗∗∗*^	0.48 (0.38; 0.58)
	SED^§^	0.42 (0.32; 0.53)	0.49 (0.39; 0.6)	0.45 (0.35; 0.56)

CK-MB	EET	0.54 (0.45; 0.62)	0.69 (0.6; 0.75)^*∗∗*^	0.74 (0.69; 0.79)^*∗∗∗*^
	SED^§/$$$^	0.27 (0.21; 0.35)^$$^	0.27 (0.20; 0.36)^$$$^	0.44 (0.35; 0.53)^*∗∗*/$$^

FABP, heart	EET	0.27 (0.2; 0.36)	0.67 (0.59; 0.73)^*∗∗∗*^	0.68 (0.6; 0.75)^*∗∗∗*^
	SED^§^	0.25 (0.19; 0.34)	0.44 (0.36; 0.54)^*∗∗∗*/$^	0.67 (0.54; 0.77)^*∗∗∗*^

IL-6	EET	0.26 (0.2; 0.34)	0.81 (0.74; 0.85)^*∗∗∗*^	0.44 (0.34; 0.54)^*∗*^
	SED^§^	0.36 (0.26; 0.48)	0.64 (0.54; 0.73)^*∗∗∗*^	0.50 (0.39; 0.61)

MMP-2	EET	0.52 (0.42; 0.61)	0.73 (0.65; 0.79)^*∗∗∗*^	0.61 (0.52; 0.69)^*∗*^
	SED^§§/$$^	0.35 (0.27; 0.43)	0.38 (0.30; 0.49)^$$^	0.37 (0.29; 0.46)^$^

MMP-3	EET	0.39 (0.31; 0.48)	0.81 (0.73; 0.86)^*∗∗∗*^	0.49 (0.40; 0.58)^*∗∗*^
	SED^§§^	0.33 (0.26; 0.41)	0.57 (0.47; 0.66)^*∗∗∗*/$^	0.39 (0.3; 0.50)

PSA-f	EET	0.52 (0.42; 0.61)	0.66 (0.55; 0.74)^*∗∗*^	0.50 (0.41; 0.59)^*∗∗*^
	SED^§^	0.43 (0.34; 0.53)	0.46 (0.37; 0.56)	0.40 (0.31; 0.51)

B2M = Beta 2 Microglobulin; CA-19-9 = Cancer Antigen 19-9; CEA = Carcinoembryonic Antigen; CK-MB = creatine kinase MB; FABP, heart = fatty acid-binding protein, heart; IL-6 = interleukin-6; MMP-2 = matrix metalloproteinase-2; PSA-f = Prostate Specific Antigen.

**Table 4:** Plasma variables of Tables 3(c) and 3(d) with significant differences between EET and SED individuals at baseline (pre). Displayed are the relative treatment effects (RTEs) and their corresponding 95% confidence intervals (CI). ^$^ *p* < 0.05 and ^$$^ *p* < 0.01 indicate significant differences between groups. EET: endurance-exercise-trained; SED: untrained/sedentary.

Variable	Group	RTE (CI) pre
AAT	EET	0.55 (0.45; 0.65)
	SED	0.35 (0.27; 0.43)^$^

B2M	EET	0.48 (0.39; 0.57)
	SED	0.26 (0.2; 0.34)^$^

CK-MB	EET	0.54 (0.45; 0.62)
	SED	0.27 (0.21; 0.35)^$$^

GH	EET	0.37 (0.31; 0.43)
	SED	0.22 (0.17; 0.29)^$^

IL-3	EET	0.63 (0.54; 0.71)
	SED	0.34 (0.26; 0.44)^$$^

IL-7	EET	0.59 (0.5; 0.67)
	SED	0.36 (0.27; 0.47)^$^

MCP-1	EET	0.52 (0.42; 0.61)
	SED	0.28 (0.21; 0.37)^$^

TNFR2	EET	0.54 (0.45; 0.63)
	SED	0.32 (0.24; 0.42)^$^

VCAM-1	EET	0.53 (0.44; 0.63)
	SED^$^	0.33 (0.26; 0.42)^$^

AAT = alpha-1-antitrypsin; B2M = Beta 2 Microglobulin; GH = growth hormone; IL-3 = interleukin-3; IL-7 = interleukin-7; IL-8 = interleukin-8; IL-10 = interleukin-10; IL-13 = interleukin-13; MCP-1 = monocyte chemotactic protein-1; TNF-*α* = tumor necrosis factor-alpha; TNFR2 = tumor necrosis factor receptor 2; VCAM-1 = Vascular Cell Adhesion Molecule-1.
